# *Borrelia burgdorferi* small lipoprotein Lp6.6 is a member of multiple protein complexes in the outer membrane and facilitates pathogen transmission from ticks to mice

**DOI:** 10.1111/j.1365-2958.2009.06853.x

**Published:** 2009-09-02

**Authors:** Kamoltip Promnares, Manish Kumar, Deborah Y Shroder, Xinyue Zhang, John F Anderson, Utpal Pal

**Affiliations:** 1 Department of Veterinary Medicine, University of MarylandCollege Park, MD 20742, USA.; 2 Virginia-Maryland Regional College of Veterinary MedicineCollege Park, MD 20742, USA.; 3 Department of Entomology, Connecticut Agricultural Experiment StationNew Haven, CT 06504, USA.

## Abstract

*Borrelia burgdorferi* lipoprotein Lp6.6 is a differentially produced spirochete antigen. An assessment of *lp6.6* expression covering representative stages of the infectious cycle of spirochetes demonstrates that the gene is solely expressed during pathogen persistence in ticks. Deletion of *lp6.6* in infectious *B. burgdorferi* did not influence *in vitro* growth, or its ability to persist and induce inflammation in mice, migrate to larval or nymphal ticks or survive through the larval-nymphal molt. However, Lp6.6-deficient spirochetes displayed significant impairment in their ability to transmit from infected ticks to naïve mice, which was restored upon genetic complementation of the mutant with a wild-type copy of *lp6.6*, establishing that Lp6.6 plays a role in pathogen transmission from ticks to mammals. Lp6.6 is a subsurface, yet highly abundant, outer membrane antigen. Two-dimensional blue native/SDS-PAGE coupled with liquid chromatography-mass spectrometry (LC-MS/MS) analysis and protein cross-linking studies independently shows that Lp6.6 exists in multiple protein complexes in the outer membrane. We speculate that the function of Lp6.6 is connected to the physiological processes of these membrane complexes. Further characterization of differentially produced membrane antigens and associated protein complexes will likely aid in our understanding of the molecular details of *B. burgdorferi* persistence and transmission through a complex enzootic cycle.

## Introduction

Lyme borreliosis is a highly prevalent tick-borne disease in the USA, Europe and many parts of Asia (Piesman and Eisen, 2008). *Borrelia burgdorferi*, the spirochetal agent of Lyme borreliosis, is maintained in nature via a complex enzootic life cycle, which involves wild rodents and ticks of the genus *Ixodes*. The pathogen contains a highly unique segmented and unstable genome (Fraser *et al.*, 1997; Casjens *et al.*, 2000) that differs significantly even from closely related pathogenic spirochetes (Porcella and Schwan, 2001). To persist in its natural transmission cycle, *B. burgdorferi* must adapt physiologically to markedly different host and vector tissue environments. A significant portion of the *B. burgdorferi* genome encodes hypothetical proteins of undefined functions (Fraser *et al.*, 1997; Casjens *et al.*, 2000), many of which are responsive to changes in the surrounding environment (Caimano *et al.*, 2007) and differentially expressed during mammalian- or arthropod-specific phases of the spirochete life cycle (de Silva and Fikrig, 1997), contributing to microbial persistence or transmission (Grimm *et al.*, 2004; Pal *et al.*, 2004; Yang *et al.*, 2004; Ramamoorthi *et al.*, 2005; Seshu *et al.*, 2006; Li *et al.*, 2007; Neelakanta *et al.*, 2007). As Lyme borreliosis may be difficult to diagnose (Aguero-Rosenfeld *et al.*, 2005) and a human vaccine to prevent the incidence of the infection is not available, characterization of antigens that impact *B. burgdorferi* persistence through the vector–host transmission cycle is important for the development of preventative and therapeutic measures against the disease.

The gene product of the *B. burgdorferi bba62* locus, annotated as a 6.6 kDa lipoprotein (Lp6.6), was originally described as an abundant, phenol-chloroform-petroleum ether-extractable low-molecular-weight lipoprotein (Katona *et al.*, 1992). Subsequent studies further identified Lp6.6 as an outer membrane (OM)-associated antigen, which appeared to be downregulated during mammalian infection (Lahdenne *et al.*, 1997). Lp6.6 is highly conserved among major *B. burgdorferi* isolates 297, N40 and B31. Lp6.6 production is regulated by alterations in the environment, such as changes in temperature (Ojaimi *et al.*, 2002) and pH (Yang *et al.*, 2001), exposure to blood in the culture medium (Tokarz *et al.*, 2004), growth in host-implanted dialysis membrane (Akins *et al.*, 1998; Brooks *et al.*, 2003; Caimano *et al.*, 2007), or in the murine host (Lahdenne *et al.*, 1997). Collectively, these studies established that *lp6.6* expression follows a prototypic ‘*ospA*’-like expression (Caimano *et al.*, 2007) where alternative sigma factor RpoS is required for the repression of both *ospA* and *lp6.6 in vivo* (Caimano *et al.*, 2005), and favours the notion that the function of Lp6.6 could be linked to the arthropod phases of the spirochete enzootic life cycle. Despite all these studies, the detailed expression profile of *lp6.6* in the spirochete infection cycle has not previously been studied and its role in *B. burgdorferi* infectivity is unknown.

Although Lp6.6 is an abundant lipoprotein in cultured spirochetes and associated with the microbial OM (Katona *et al.*, 1992; Lahdenne *et al.*, 1997), the antigen is not likely exposed on the microbial surface (Lahdenne *et al.*, 1997), and thus, may not directly participate in host–pathogen interaction. Certain cellular proteins, including small OM bacterial lipoproteins (Sklar *et al.*, 2007; Lewis *et al.*, 2008), often assemble into protein complexes (Alberts, 1998) that carry out specific roles in microbial biology including energy generation, protein assembly, lipoprotein trafficking and small molecule transport, which contributes to microbial pathogenesis and survival in the host environment (Stenberg *et al.*, 2005; Pyndiah *et al.*, 2007). As Lp6.6 is abundant in the spirochete membrane, we have assessed if Lp6.6 forms protein complexes, and determined whether Lp6.6 function is required for *B. burgdorferi* persistence through an experimental tick–mouse infection cycle. The characterization of membrane antigens that are differentially expressed during the host- or vector-specific pathogen life cycle is important for the development of novel strategies to interfere with *B. burgdorferi* transmission and prevention of Lyme borreliosis.

## Results

### Expression of *lp6.6* throughout the mouse–tick infection cycle of *B. burgdorferi*

**Fig. 1:**
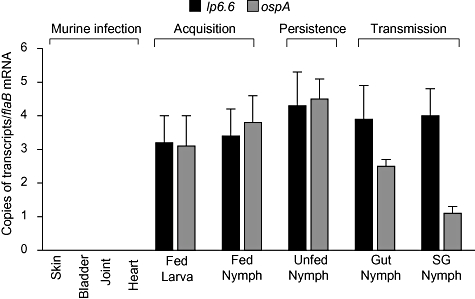
Expression of *lp6.6* and *ospA* in representative stages of *B. burgdorferi* enzootic life cycle. The relative expression levels of *lp6.6* during murine infectivity, acquisition and persistence in larval and nymphal ticks, and transmission through infected nymphs are analysed. Expression of *ospA* was also assessed in the tick samples. Transcript levels of *lp6.6* and *ospA* were measured using quantitative RT-PCR and presented as copies of target transcripts per copy of the *flaB* transcript. RNA was isolated from mice at 2 weeks after *B. burgdorferi* infection, from larvae and nymphs at 3 days of feeding on *B. burgdorferi-*infected mice or from freshly molted unfed infected nymphs, and from gut and salivary glands (SG) of *B. burgdorferi*-infected nymphs feeding on naïve mice at 2 days of feeding. The bars represent the mean values and the error bars represent the SEM values from six quantitative RT-PCR analyses of two independent murine-tick infection experiments.

*Borrelia burgdorferi bba62* encodes for a major membrane lipoprotein, annotated as Lp6.6, that appears to be downregulated during mammalian infection (Lahdenne *et al.*, 1997). To understand the role of Lp6.6 in the spirochete enzootic life cycle, we assessed the temporal and spatial expression of *lp6.6* throughout representative stages of the infectious cycle of *B. burgdorferi* using ticks and murine hosts. C3H/HeN mice were infected with *B. burgdorferi* and skin, joint, heart and bladder samples were collected following 2 weeks of infection. Larval and nymphal ticks were fed on parallel groups of mice following 2 weeks of infection (25 ticks per mouse) and engorged ticks were isolated at 3 days of feeding. One group of fed intermolt larvae were allowed to molt to nymphs and analysed as infected unfed nymphs. Another parallel group of unfed infected nymphs were allowed to feed on naïve mice (25 ticks per mice), and their gut and salivary glands were isolated at 2 days of feeding. Total RNA was prepared from murine and tick samples, and subjected to quantitative reverse transcription polymerase chain reaction (qRT-PCR) analysis to measure *lp6.6* transcripts. As *lp6.6* has been speculated to follow ‘*ospA*’-like expression (Caimano *et al.*, 2007), the same set of tick samples were also assessed for *ospA* transcripts. The results supported a previous study (Lahdenne *et al.*, 1997) showing that *lp6.6* transcripts are undetectable in infected murine tissues (Fig. 1). *lp6.6* expression is upregulated as soon as *B. burgdorferi* enters ticks, either larvae or nymphs, from infected mice and, similarly to *ospA*, remains highly expressed throughout the tested stages of the spirochete life cycle in the vector. However, unlike *ospA*, the levels of *lp6.6* transcripts were abundant during transmission of *B. burgdorferi* from ticks to the murine host.

### Generation and characterization of Lp6.6-deficient *B. burgdorferi*

**Fig. 2:**
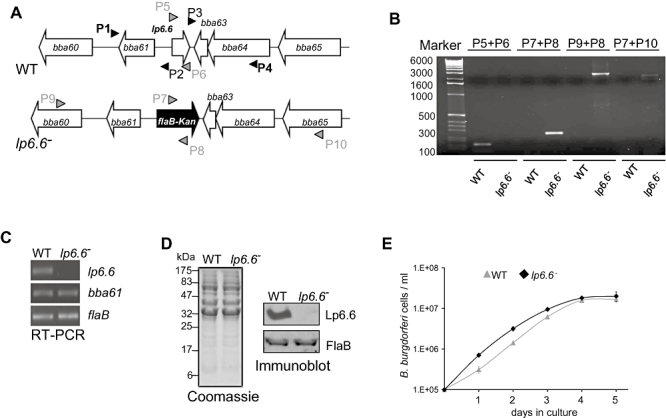
Construction and analysis of the *lp6.6* mutant *B. burgdorferi*. A. Schematic drawings of the wild-type (WT) and the *lp6.6* mutant (*lp6.6*^−^) isolates at the *lp6.6* locus. Genes *bba60*–*bba65* (white box arrows) and the kanamycin-resistance cassette driven by the *B. burgdorferi flaB* promoter (*flaB-Kan*, black box arrow) are indicated. Primers P1–P4 (black arrowheads) were used to amplify 5′ and 3′ arms for homologous recombination and ligated on either side of the *flaB-Kan* cassette as detailed in the text. B. Desired integration of the mutagenic construct, *flaB-Kan*, in *lp6.6* genomic locus. Primers 5–10 (grey arrowheads, positions indicated in A) were used for DNA amplifications in wild type or *lp6.6* mutant and subjected to gel electrophoresis. The combination of primers used for PCR is indicated at the top, and migration of the DNA ladder is shown on the left. C. RT-PCR analysis of *lp6.6* deletion and the polar effect of mutagenesis. Isolated cDNA was used to amplify regions within *lp6.6*, *flaB*, and the gene downstream of *lp6.6* locus (*bba61*) and visualized on a gel. D. Protein analysis of *B. burgdorferi*. Equal amounts of proteins from wild type and *lp6.6* mutant were separated on a SDS-PAGE gel, and either stained with Coomassie blue (left) or transferred onto a nitrocellulose membrane and probed with Lp6.6 and FlaB antibodies (right). Migration of protein standards is shown to the left in kDa. E. Growth curves for the wild-type and *lp6.6* mutant spirochetes. Spirochetes were diluted to a density of 10^5^ cells ml^−1^, grown at 33°C in BSK-H medium, and counted under a dark-field microscope every 24 h using a Petroff–Hausser cell counter. Differences between wild type and *lp6.6* mutant numbers were insignificant at all times of growth (*P* > 0.05).

To further study the role of Lp6.6 in the *B. burgdorferi* life cycle, we created Lp6.6-deficient *B. burgdorferi*. An infectious *B. burgdorferi* isolate was used to create an isogenic mutant by exchanging the *lp6.6* (*bba62*) open reading frame with a kanamycin-resistance cassette via homologous recombination (Fig. 2A). Out of five transformed clones that grew in antibiotic-containing media, one clone was selected through polymerase chain reaction (PCR) analysis of the desired integration of the antibiotic cassette (Fig. 2B) and retention of all endogenous plasmids present in the parental isolate (data not shown). RT-PCR analysis showed that the mutant failed to produce *lp6.6* mRNA, and that mutagenesis did not impose polar effects on the transcription of the immediate upstream gene *bba61* (Fig. 2C). Transcription of the downstream gene, *bba63*, which has a small open reading frame (126 nucleotides) with a highly repetitive DNA sequence, could not be evaluated; however, a polar effect is unlikely due to its opposite transcriptional direction to that of *lp6.6*. The protein profile of the *lp6.6* mutant was similar to that of the wild-type spirochete (Fig. 2D, left), and the mutant failed to produce Lp6.6 protein (Fig. 2D, right). Compared with parental isolates, the *lp6.6* mutant displayed a similar growth rate when cultured *in vitro* at 33°C (Fig. 2E) or at 23°C (data not shown).

### *lp6.6* mutants remain infectious in mice

**Fig. 3:**
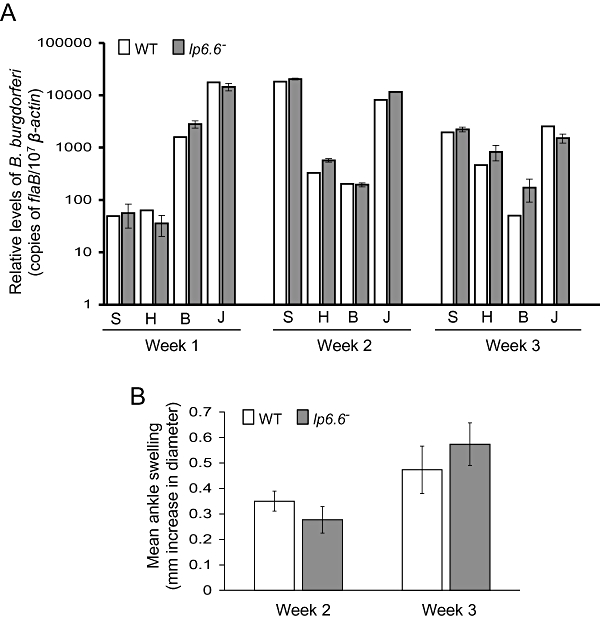
*lp6.6* mutant *B. burgdorferi* retains murine infectivity. A. The *B. burgdorferi* burdens in multiple tissues of infected mice are shown. Mice (five animals per group) were infected with either the wild-type or the *lp6.6* mutant *B. burgdorferi* and spirochete burdens were analysed in skin (S), heart (H), bladder (B) and joint (J) samples by measuring copies of *B. burgdorferi flaB* RNA at weeks 1, 2 and 3 following infection. Amounts of murine *β-actin* mRNA were determined in each sample and used to normalize the quantities of spirochete RNA. The bars represent the mean values and the error bars represent the SEM values from four quantitative RT-PCR analyses from two independent infection experiments. Differences between wild-type and *lp6.6* mutant burdens were statistically insignificant in any tissue or time point measured (*P* > 0.05). B. Severity of joint swelling in *B. burgdorferi-*infected mice. Groups of mice (five animals per group) were separately infected with wild type or *lp6.6* mutant and inflammation was evaluated by the assessment of joint swelling following weeks 2 and 3 of spirochete challenge using a digital caliper. The bars represent the mean values and the error bars represent the SEM values from two independent infection experiments. Wild type and *lp6.6* mutant induced similar joint swelling (*P* > 0.05).

To examine whether the lack of *lp6.6* influences *B. burgdorferi* infectivity in mammals, C3H/HeN mice (five animals per group) were inoculated intradermally with equal numbers of wild-type or *lp6.6* mutant *B. burgdorferi* (10^5^ spirochetes per mouse). *B. burgdorferi* infection was assessed by qRT-PCR analysis of viable pathogen burden in murine skin, heart, bladder and joint samples isolated after 1, 2, 3 and 12 weeks of infection. Murine spleen samples were collected at the same time points and spirochete viability was further assessed by culture analysis. Results indicated insignificant differences between burdens of wild type and *lp6.6* mutants in a diverse range of tissues (skin, heart, bladder and joint) and phases of infection, between 1 and 3 weeks (Fig. 3A) or even at 12 weeks of infection (data not shown). Similarly, both the mutant and wild-type spirochetes were isolated by culture of infected spleen (data not shown). As suggested by the similar pathogen burdens, mice infected with wild-type or *lp6.6* mutant *B. burgdorferi* developed similar levels of disease, as evaluated by the development of ankle swelling (Fig. 3B) and histopathological signs of arthritis (data not shown)*.*

### *lp6.6* mutants display no defects in acquisition and persistence in ticks

**Fig. 4:**
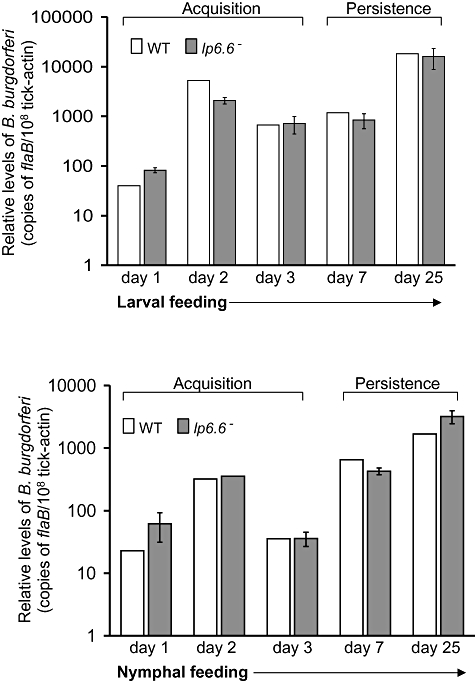
*lp6.6* mutant *B. burgdorferi* displays no defects in its ability to enter and persist in ticks. Mice were infected with *B. burgdorferi* (two mice per group) and following 12 days of infection, naïve *I. scapularis* larvae or nymphs (25 ticks per group) were allowed to feed on mice, and *B. burgdorferi* burdens in ticks were analysed at the indicated time intervals following feeding by measuring copies of the *B. burgdorferi flaB* RNA. Amounts of tick *β-actin* mRNA were determined in each sample and used to normalize the quantities of spirochete RNA. The bars represent the mean values and the error bars represent the SEM values from two independent experiments. Burdens of wild type and *lp6.6* mutant are similar at all time points (*P* > 0.05).

We next determined whether Lp6.6-deficient *B. burgdorferi* could efficiently migrate from the infected murine host to the arthropod vector and then persist within *Ixodes scapularis*. To achieve this, *Ixodes* larval and nymphal ticks (25 ticks per group) were allowed to engorge on parallel groups of mice after 12 days of infection with wild-type or *lp6.6* mutant *B. burgdorferi*. The ticks were collected following 1, 2 and 3 days of feeding and also during intermolt stages, 7 and 25 days after engorgement. The samples were subjected to qRT-PCR analyses to assess viable spirochete burdens. The results indicated no significant difference in the burdens of *lp6.6* mutant and wild-type isolates in any stages of spirochete acquisition and persistence in larval or nymphal ticks (Fig. 4). Overall, these observations suggest that despite highly detectable *lp6.6* expression (Fig. 1), Lp6.6 is not required for the acquisition or persistence of *B. burgdorferi* in ticks.

### *lp6.6* mutant displays a phenotypic defect in transmission from infected ticks to naïve mice

**Fig. 5:**
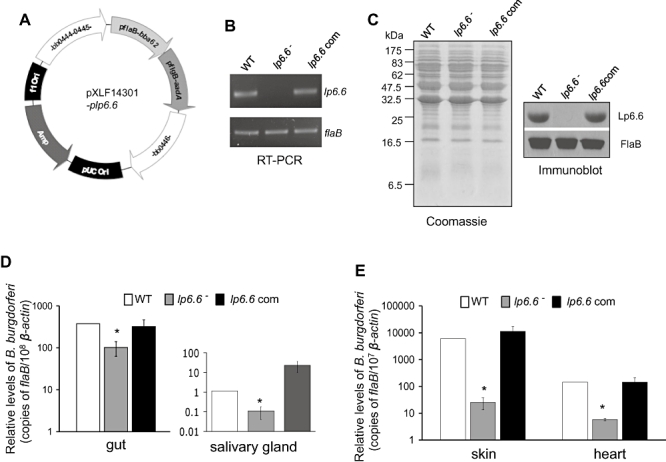
Complementation of *lp6.6* mutant *B. burgdorferi* with *lp6.6* restores the phenotypic defects of the mutant to transmit from ticks to mice. A. Construction of plasmid DNA pXLF14301-*plp6.6* for *cis*-integration of the *lp6.6* gene. The open reading frame of *lp6.6* gene was fused with the *flaB* promoter and cloned into the shuttle vector pKFSS1 that houses the streptomycin resistance gene (*aadA*) under *B. burgdorferi flgB* promoter. The *flaB* promoter–*lp6.6* gene fusion and *aadA* cassette was finally cloned into pXLF14301, which contains DNA fragments for homologous recombination and integration of the complemented gene in the *B. burgdorferi* genome. B. RT-PCR analysis of the *lp6.6* transcripts. Total RNA was isolated from either the wild-type (WT), *lp6.6* mutant (*lp6.6*^−^) or *lp6.6-*complemented *B. burgdorferi* (*lp6.6* com), converted to cDNA, then subjected to PCR analysis with *flaB* and *lp6.6* primers, and analysed on an agarose gel. C. Restoration of Lp6.6 protein by the complemented *B. burgdorferi*. Lysates of *B. burgdorferi* were separated on a SDS-PAGE gel, which was either stained with Coomassie blue or transferred to nitrocellulose membrane, and probed with antiserum against Lp6.6 or FlaB. D. *B. burgdorferi* transmission through infected ticks. *B. burgdorferi*-infected nymphs were generated by feeding larvae on mice infected with wild-type and genetically manipulated *B. burgdorferi* (two mice per group) as described in the text. Newly molted *B. burgdorferi-*infected nymphs were allowed to feed on naïve mice (five ticks per mouse, five animals per group). *B. burgdorferi* burdens were assessed in dissected tick guts (left) or salivary glands (right) at 2 days of feeding by measuring copies of the *B. burgdorferi flaB* RNA and normalized against tick *β-actin* RNA levels. E. *B. burgdorferi* transmission from infected ticks to mice. *B. burgdorferi*-infected nymphs were fed on mice as described in (D) and spirochete levels were assessed in the indicated murine tissues at 7 days of tick feeding. The bars represent the mean values and the error bars represent the SEM values from two independent animal infection experiments. Burdens of wild type and *lp6.6-*complemented mutants are significantly higher than corresponding levels of *lp6.6* mutants (*P < 0.05).

We finally assessed whether Lp6.6 is required for *B. burgdorferi* transmission from ticks to mice. To mimic the natural transmission process, we first generated *B. burgdorferi-*infected nymphs by allowing larvae to acquire spirochetes from infected mice and then molt into *B. burgdorferi-*infected nymphs in the laboratory. When infected nymphs were allowed to feed on naïve C3H mice, the burden of *lp6.6* mutants declined in ticks at 2 days of feeding. To rule out the possibility that the potential phenotypic defects of the mutant during the transmission process was the result of anomalous effects of genetic manipulation, we sought to complement the *lp6.6* mutant spirochetes with a wild-type copy of the *lp6.6* gene, and use this isolate in tick–mouse transmission studies. For stable integration of the complemented construct, *lp6.6*, along with an antibiotic resistance cassette, was inserted into an intergenic chromosomal locus in *B. burgdorferi*. As our efforts for complementation of *lp6.6* mutant involving the native promoter failed to yield any transformants, we sought to complement the mutant using the *B. burgdorferi flaB* promoter as described (Yang *et al.*, 2009). To accomplish this, we first fused the open reading frame of *lp6.6* with the *flaB* promoter and cloned it into the pKFSS1 vector (Frank *et al.*, 2003), which carries an *aadA* streptomycin resistance cassette. A DNA element encompassing the *flaB–lp6.6* fusion, along with the *aadA* cassette, was then assembled into the recombinant plasmid pXLF14301-*plp6.6* (Fig. 5A) and integrated into *B. burgdorferi* genome via allelic exchange. Five transformants that grew in BSK medium containing both kanamycin and streptomycin were isolated. PCR analyses further identified one of the *lp6.6*-complemented clones, which retained the endogenous plasmids similar to those present in the parental isolate (data not shown). RT-PCR and immunoblotting showed that the *lp6.6*-complemented isolate produced both *lp6.6* mRNA (Fig. 5B) and Lp6.6 protein (Fig. 5C).

We then compared the ability of the wild-type, *lp6.6* mutant and complemented spirochetes to transmit from infected ticks to naïve mice. Naturally infected nymphs were generated by allowing larval ticks to acquire spirochetes from mice infected with wild-type or genetically manipulated *B. burgdorferi*. Infected ticks were allowed to feed on naïve C3H mice (five ticks per mouse), collected at 1, 2 and 4 days of feeding and viable spirochete burdens were assessed by qRT-PCR analysis. The results showed that, while differences between the burden of wild-type and mutant spirochetes were insignificant in ticks collected at 1 or 4 days of feeding (data not shown), the levels of Lp6.6-deficient *B. burgdorferi*, compared with wild-type and *lp6.6-*complemented spirochetes, were significantly lower in the gut (Fig. 5D, left) and salivary glands (Fig. 5D, right) collected at 2 days of feeding. Spirochete burdens in mice were assessed by qRT-PCR at an early time point of infection, 7 days of tick feeding, which indicated that the *lp6.6* mutant is significantly impaired in transmission to mice (Fig. 5E), whereas *lp6.6-*complemented isolates were transmitted to the host at comparable levels to the wild type. In spite of impaired transmission, the *lp6.6* mutant was recovered by culture analyses of infected murine tissues (data not shown) suggesting that while Lp6.6 is not essential, it facilitates *B. burgdorferi* transmission through feeding ticks to the murine host.

### Lp6.6 is a member of multiple protein complexes in the OM

**Fig. 6:**
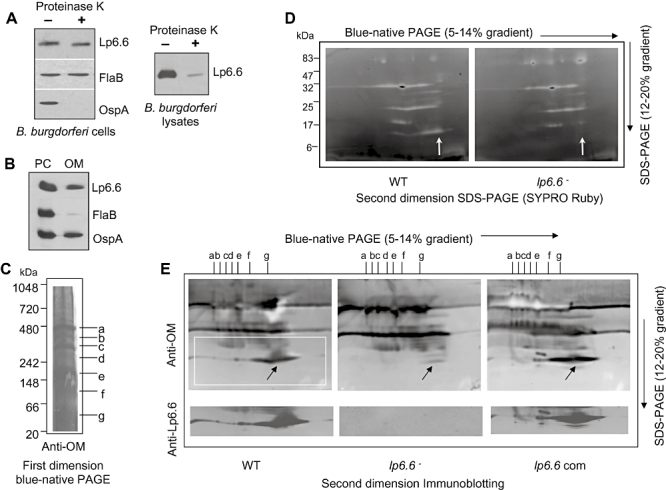
Lp6.6 is localized in the *B. burgdorferi* outer membrane (OM) with subsurface topology and is a member of multiple membrane protein complexes. A. Lp6.6 is insensitive to proteinase K-mediated degradation of *B. burgdorferi* surface proteins*.* Viable spirochetes were incubated with (+) or without (−) proteinase K for the removal of protease-sensitive surface proteins and processed for immunoblot analysis using Lp6.6 antibodies (left). *B. burgdorferi* OspA and FlaB antibodies were utilized as controls for surface-exposed and subsurface proteins respectively. Lp6.6 is highly sensitive to proteinase K digestion in lysed preparations of *B. burgdorferi* (right). B. Cellular Lp6.6 is found in the isolated OM of cultured spirochetes. *B. burgdorferi* protoplasmic cylinders (PC) and OM were separated by sucrose density gradient centrifugation and equal amounts of protein from two subcellular fractions were separated by SDS-PAGE, and immunoblotted with Lp6.6, OspA and FlaB antiserum. C. OM protein complexes separated by one-dimensional blue-native (BN) gel. OM protein complexes from wild-type *B. burgdorferi* were separated in 5–14% BN-PAGE as described in the text, transferred onto a nitrocellulose membrane and probed with OM antibodies. Identified protein complexes were labelled from a to g. D. Two-dimensional analysis of OM protein complexes in wild type and *lp6.6* mutant. Strips of BN-PAGE were excised and placed on the top of the 12–20% denaturing gels and stained by SYPRO Ruby. The vertical arrow indicates the position of Lp6.6. E. Immunoblots of 2D-BN/SDS-PAGE from isolated OM from wild-type, *lp6.6* mutant and *lp6.6*-complemented (com) isolates using antibodies specific for OM and Lp6.6. The arrow indicates the bands assigned to Lp6.6.

Although the above studies indicate that Lp6.6 plays a role in spirochete transmission, the function of Lp6.6 in the biology of *B. burgdorferi* is unknown. Lp6.6 expression in cultured spirochetes is highly sensitive to environmental alterations and is differentially expressed *in vivo* (Fig. 1); however, the antigen lacks surface exposure (Fig. 6A) and thus is unlikely to be directly involved in pathogen interaction with the surrounding environment. While Lp6.6 was previously identified as an OM lipoprotein (Katona *et al.*, 1992; Lahdenne *et al.*, 1997), we assessed the precise cellular distribution of Lp6.6. To accomplish this, cultured spirochetes were separated into two major fractions, outer membrane vesicle (OMV) and protoplasmic cylinder (PC), and subjected to immunoblot analyses with anti-Lp6.6, -OspA and -FlaB antibodies. While FlaB was almost undetectable in the OM, both OspA and Lp6.6 were abundant in the OM (Fig. 6B).

**Fig. 7:**
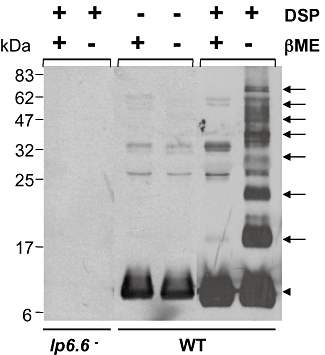
Outer membrane Lp6.6 forms high-molecular-weight complexes in the presence of a protein cross-linker. Outer membrane proteins were isolated from wild-type and Lp6.6-deficient *B. burgdorferi* and subjected to cross-linking with dithiobis(succinmidylpropionate) (DSP). DSP-treated samples were further incubated in the presence or absence of β-mercaptoethanol to cleave the DSP-mediated cross-linking, separated on SDS-PAGE gels and immunoblotted with Lp6.6 antibodies. Arrows indicated formation of multiple high-molecular-weight cross-linked protein complexes containing Lp6.6. Arrowhead donates non-cross-linked free Lp6.6 proteins.

As Lp6.6 is abundant in the OM, we assessed whether the antigen participates in protein complexes, which contribute to membrane physiology and microbial pathogenesis. To explore this, we used two-dimensional blue native (2D-BN)/SDS-PAGE combined with mass spectrometry and protein cross-linking approaches to determine whether Lp6.6 is a member of membrane protein complexes. OM proteins from wild-type *B. burgdorferi* were separated using one-dimensional BN-PAGE and immunoblotting with anti-OM antibodies identified seven major protein complexes (Fig. 6C, labelled as a–g). The approximate molecular masses of the protein complexes ranged from 50 to 480 kDa. To identify if Lp6.6 participates in protein complex formation, OMV were isolated from both wild-type and genetically manipulated *B. burgdorferi*, separated in the first dimension through BN-PAGE and further analysed in the second dimension by SDS-PAGE, which resolved individual complexes into vertical ‘channels’, enabling visualization of the individual constituents of the complexes. Gels were either stained with SYPRO Ruby (Fig. 6D) or immunoblotted with OM or Lp6.6 antibodies (Fig. 6E), which indicated that Lp6.6 is a member of protein complexes. Liquid chromatography-mass spectrometry (LC-MS/MS) analysis further confirmed the presence of Lp6.6 in multiple protein complexes, where each complex (marked as a–g, Fig. 6C) contained matching Lp6.6 peptides with a minimum of 38% coverage of the protein. Unlike *lp6.6* mutant, both wild-type and *lp6.6*-complemented *B. burgdorferi* displayed protein complexes containing Lp6.6 (Fig. 6E). To obtain independent experimental evidence that Lp6.6 forms membrane protein complexes, isolated OM from wild-type and Lp6.6-deficient *B. burgdorferi* was subjected to protein cross-linking analysis using dithiobis(succinmidylpropionate) (DSP), an amine-reactive, homobifunctional cross-linker with a spacer arm of 12 Å length, which contains a disulphide bond that can be cleaved with reducing agents, such as β-mercaptoethanol (βME). Immunoblot analysis with Lp6.6 indicated that DSP readily cross-linked Lp6.6 in OM isolated from wild-type spirochetes, where multiple high-molecular-weight cross-link products containing Lp6.6 were obtained (arrows, Fig. 7). Although the components of these Lp6.6-containing complexes, either different sizes of Lp6.6 polymers or multiprotein units, remain indistinguishable, DSP-mediated Lp6.6 cross-linking was specific as the products were completely dissociated into free proteins in the presence of βME (arrowhead, Fig. 7). Cross-linked products were also undetectable in OM isolated from *lp6.6* mutant spirochetes (left lanes, Fig. 7).

## Discussion

The present study establishes that *in vivo* expression of a small lipoprotein of *B. burgdorferi*, Lp6.6, is confined to the vector phases of the pathogen life cycle, and that while Lp6.6 is not essential for pathogen persistence, it facilitates the transmission of spirochetes from ticks to the murine host. We show that Lp6.6 is a constituent member of multiple protein complexes in the spirochete OM, indicating that the function of Lp6.6 may be related to as yet undefined aspects of membrane physiology.

*Borrelia burgdorferi* persists in a diverse range of tissue environments as it cycles between the arthropod vector and the mammalian host. Spirochetes alter their gene expression in response to alterations in *in vitro* growth conditions (Ojaimi *et al.*, 2002; Revel *et al.*, 2002; Brooks *et al.*, 2003; Tokarz *et al.*, 2004; Caimano *et al.*, 2007) and in different phases of microbial persistence in mammalian hosts and ticks (Narasimhan *et al.*, 2002; 2003). Many of these transcriptional modulations lead to changes in the antigenic composition of *B. burgdorferi*, including OM lipoproteins (Hefty *et al.*, 2002), which directly face the surrounding environment and likely play important roles in microbial adaptation to a given microenvironment. A significant fraction of the *B. burgdorferi* genome encodes for proteins with export signals (Casjens *et al.*, 2000; Schulze and Zückert, 2006), many of which are abundant in the membrane and produced *in vivo* (Nowalk *et al.*, 2006; Barbour *et al.*, 2008) and, thus, likely involved in spirochete adaptation. Lp6.6 is subsurface lipoprotein (Katona *et al.*, 1992) that lacks a detectable antibody response in infected hosts (Lahdenne *et al.*, 1997). Previous studies determined that overexpression of vector-specific borrelial genes in mice, such as *ospA*, by genetically manipulated isolates, induces robust and specific borreliacidal antibodies that severely impact *B. burgdorferi* persistence (Strother *et al.*, 2007; Xu *et al.*, 2008). However, continual *lp6.6* expression by complemented mutants within the murine host did not significantly influence spirochete persistence (data not shown). Many of the borrelial membrane proteins that are differentially expressed in the vector or in hosts, such as OspA/B, BmpA/B, DbpA/B and CRASP-related proteins, are members of a common operon or paralogous gene family (Casjens *et al.*, 2000), and in many instances are co-transcribed (Dobrikova *et al.*, 2001; El-Hage and Stevenson, 2002)*.* Although *lp6.6* is a unique gene, its expression pattern mostly resembles that of *ospA*, and is likely regulated by similar environmental signals and genetic regulatory networks (Caimano *et al.*, 2007). However, *lp6.6* does not downregulate in parallel with *ospA* during nymphal feeding, raising the possibility that, at least during transmission, *lp6.6* expression might be governed by an unknown regulatory pathway*.* Nevertheless, the consistent expression of *lp6.6* within ticks, which is analogous to other borrelial genes important in the vector, such as *ospA* and *ospB* (Yang *et al.*, 2004; Neelakanta *et al.*, 2007), indicates that Lp6.6 function is likely important for the spirochete life cycle in ticks. While *lp6.6* mutant did not display detectable growth deficiencies *in vitro*, transient defects in persistence within feeding-infected ticks were apparent (Fig. 5D). This is the phase at which spirochetes dramatically increase in number (de Silva and Fikrig, 1995) and disseminate from feeding ticks to the host (Piesman *et al.*, 1987; Piesman, 1993; Ohnishi *et al.*, 2001). The detection of lower numbers of *lp6.6* mutants in the tick gut due to possibly more efficient transmission is unlikely, as burdens of *lp6.6* mutants that exited the gut and migrated either to the salivary glands (Fig. 5D, right) or to the murine host (Fig. 5E) were significantly lower than wild-type and complemented isolates. Lp6.6 thus serves an unknown function during the transmission of spirochetes, which involves a series of carefully orchestrated yet poorly defined events including timely escape through gut epithelia and potential peritrophic barriers, dissemination via the haemolymph, invasion of the salivary gland and transfer to the mammalian dermis (Fikrig and Narasimhan, 2006). Spirochete proteins that are differentially produced in ticks, including Lp6.6 and other proteins regulated by the RpoN–RpoS pathway (Fisher *et al.*, 2005), might play a role in *B. burgdorferi* transmission from ticks as well as in establishment of early host infection.

In cellular membranes, multiple proteins assemble into distinct protein complexes (Alberts, 1998), which perform specific roles in membrane physiology and contribute to microbial virulence (Stenberg *et al.*, 2005; Pyndiah *et al.*, 2007). We show that Lp6.6, although differentially expressed in the spirochete life cycle, is one of the prominent constituent members of all OM protein complexes*.* Both 2D-BN/SDS-PAGE and protein cross-linking analyses identified Lp6.6 as a participant in multiple protein complexes, with the highest abundance in low-molecular-weight complexes. However, we cannot rule out the possibility that the observed chemical cross-linking of Lp6.6 could be due to the abundance of antigen in the OM, and thus, opportunistic cross-linking to other membrane proteins. The appearance of Lp6.6-containing protein complexes in 2D-BN/SDS-PAGE as a continual smear is commonly observed in protein complexes of other bacteria (Krall *et al.*, 2002; Stenberg *et al.*, 2005; Pyndiah *et al.*, 2007). The occurrence of protein complexes in the bacterial OM and their roles in pathogen biology vary among Gram-negative bacteria. For example, *Helicobacter pylori* OM contains only two multiprotein complexes (Pyndiah *et al.*, 2007), fumarate reductase and urease enzyme complexes, composed of 10 individual proteins and primarily involved in the energy metabolism, which may not be essential for *H. pylori* survival *in vitro* but are required for pathogen colonization in the host gut (Ge *et al.*, 2000). *Escherichia coli* OM, on the other hand, contains nine homo- or hetero-oligomeric complexes composed of a total of 12 participating proteins and each complex carries a specific function related to general diffusion, drug transport, stationary-phase adaptation and biogenesis or integrity of the OM (Stenberg *et al.*, 2005). We have identified seven protein complexes in the OM of wild-type spirochetes, which are also maintained in *lp6.6* mutants. Therefore, Lp6.6 is probably not a core member of the protein complexes required for their assembly, but may provide an auxiliary role, for example, by maintaining complex stability, as shown for a small OM lipoprotein in *E. coli* (Sklar *et al.*, 2007) and *Salmonella enterica* (Lewis *et al.*, 2008). Similarly, a non-essential yet supportive role of Lp6.6 in the spirochete infection cycle was also shown by our mutagenesis studies.

The *B. burgdorferi* membrane undergoes dramatic antigenic variation *in vivo*, due to sequence-specific recombination (Coutte *et al.*, 2009) and differential gene expression (Schwan, 2003) as it cycles through the tick–rodent infection cycle. Here we show at least one of these differentially produced antigens, Lp6.6, to be a member of the membrane protein complexes of cultured organisms. We do not know the biogenesis, stability or contributions to spirochete infectivity of these complexes. Future characterization of the spirochete membrane protein complexome, identification of interacting proteins besides Lp6.6, and their roles in pathogen biology and infectivity may provide new insights into the molecular details of *B. burgdorferi* survival through a complex enzootic cycle.

## Experimental procedures

### *B. burgdorferi*, ticks and mice

A clonal and low-passage infectious isolate of *B. burgdorferi*, B31-A3 (Elias *et al.*, 2002), was used throughout this study. Four- to six-week-old C3H/HeN mice were purchased from the National Institutes of Health. *I. scapularis* ticks used in this study originated from a colony that is maintained in the laboratory (Coleman *et al.*, 2008). All animal experiments were performed in accordance with the guidelines of the Institutional Animal Care and Use Committee and Institutional Bio-safety Committee of the University of Maryland, College Park.

### Polymerase chain reaction

The oligonucleotide sequences for each of the primers used in specific PCR reactions are indicated in Table S1. Total RNA was isolated using TRIzol reagent (Invitrogen), reverse transcribed to cDNA (AffinityScript, Stratagene), and RT-PCR or qRT-PCR analysis was performed as described (Coleman *et al.*, 2008). Expression of *lp6.6* was analysed in groups of five C3H/HeN mice (10^5^ spirochetes per mouse) at 2 weeks after infection or in larval and nymphal ticks that fed on mice infected for 2 weeks (25 ticks per mouse) as detailed (Coleman *et al.*, 2008). For *lp6.6* and *ospA* expression analyses during spirochete acquisition and persistence in ticks, whole larva and nymph were analysed at 3 days of feeding or as freshly molted infected unfed nymphs. For expression analysis during transmission, *B. burgdorferi-*infected nymphs were fed on naïve mice (25 ticks per mice, two mice per group), and dissected guts and salivary glands were analysed at 2 days of feeding as described (Pal *et al.*, 2004; Coleman *et al.*, 2008). The levels of *B. burgdorferi lp6.6* transcript in tick and mouse samples were normalized against *flaB* transcripts in the qRT-PCR reaction as detailed (Coleman *et al.*, 2008).

### Production of recombinant Lp6.6, generation and characterization of Lp6.6 antiserum

The *lp6.6* gene was cloned into pGEX-4T-2 (Amersham-Pharmacia Biotech) using specific primers (Table S1) and the recombinant protein Lp6.6 without the N-terminal leader sequence was produced in *E. coli*. Expression and purification of the glutathione *S*-transferase (GST) fusion protein were performed as described previously (Pal *et al.*, 2008a). Generation of polyclonal murine antiserum against recombinant Lp6.6–GST fusion protein and assessment of titre and specificity of the antisera, using ELISA and immunoblotting, were performed as described (Pal *et al.*, 2008a).

### Proteinase K accessibility assay

Proteinase K accessibility assays were performed as detailed (Coleman *et al.*, 2008). Briefly, spirochetes (1 × 10^8^) were suspended in phosphate-buffered saline (PBS) and split into two equal volumes. One aliquot received 200 mg ml^−1^ of proteinase K (Sigma) while the other aliquot received an equal volume of PBS without the enzyme. Both aliquots were incubated for 20 min, treated with phenylmethylsulphonylfluoride (Sigma), pelleted and re-suspended in PBS for immunoblot analysis using antibodies against Lp6.6, FlaB or OspA. To assess whether Lp6.6 is sensitive to protease digestion, additional batches of spirochetes (1 × 10^8^) were sonicated, and equal amounts of solubilized proteins were incubated in the absence and presence of proteinase K, and immunoblotted with Lp6.6 antibodies.

### Purification of OM, generation and characterization of anti-OM antiserum

Isolation of the OM of *B. burgdorferi* was performed as described (Skare *et al.*, 1995). Briefly, 5 × 10^10^ to 1 × 10^11^*B. burgdorferi* cells were washed in PBS, pH 7.4, supplemented with 0.1% BSA. The cells were re-suspended in ice-cold 25 mM citrate buffer pH 3.2 containing 0.1% BSA and incubated on a rocker at room temperature for 2 h. OMV were released from whole cells and were isolated from protoplasmic cylinder (PC) by using sucrose density gradient centrifugation. The isolated OM was monitored for purity by immunoblotting using antibodies against OspA and FlaB. Immunoblotting results showed an enrichment of OspA in OM proteins and only with minor cross-contamination of FlaB. Generation of polyclonal OM antisera in rabbits, assessment of titre and specificity of the antisera using ELISA and immunoblotting were performed as described (Pal *et al.*, 2008b).

### 2D-BN/SDS-PAGE and immunoblotting

Analysis of OM proteins was performed under native conditions by BN-PAGE as described (Schagger and von Jagow, 1991). Isolated membranes were solubilized with β-dodecyl maltoside (DM) (DM/protein = 20 w/w) and protein complexes were analysed at 4°C in 5–14% polyacrylamide gel. Native protein markers (Invitrogen) were used to estimate the size of protein complexes in BN gels. Subunit composition of the protein complexes was assessed by electrophoresis in denaturing 12–20% linear gradient polyacrylamide gels containing 7 M urea (Komenda *et al.*, 2002). Pre-stained protein markers (New England BioLabs) were used for estimation of apparent molecular masses of proteins in SDS-PAGE gels. The whole lanes from the BN gel were excised, and either transferred onto nitrocellulose membrane for immunodetection with antibodies against OM or incubated for 30 min in 25 mM Tris/HCl, pH 7.5, containing 1% SDS (w/v) and 1% DTT (w/v), and placed on the top of the denaturing gel. Proteins separated in the gel were either stained by SYPRO Ruby (Invitrogen) or transferred onto nitrocellulose membrane for immunodetection with antibodies against OM and Lp6.6. Protein signals were visualized using ECL Western Blotting Detection Reagent (Amersham, UK).

### Liquid chromatography-mass spectrometry (LC-MS/MS)

Samples for tandem mass spectrometry were prepared by tryptic in-gel digestion of excised protein bands as described (Williams and Stone, 1997). Briefly, protein complexes from one-dimensional BN-PAGE were excised and put into 1.5 ml tubes that were pre-washed with 500 μl of buffer containing 0.1% TFA and 60% acetonitrile (ACN). Gel bands were successively washed with 250 μl of 50% ACN, buffer containing 50% ACN, 50 mM NH_4_HCO_3_ and 10 mM NH_4_HCO_3_, dried with a speedvac and digested with sequencing grade trypsin (0.1 mg ml^−1^), further extracted with 50 μl of 50% ACN and 5% TFA and finally dried with a speedvac. LC-MS/MS analyses and protein identification were performed at the Proteomics Core Facility of College of Chemical and Life Sciences, University of Maryland. Briefly, tryptic digests were injected onto a Zorbax SB300 C18 column (1.0 × 100 mm, Agilent Technologies) connected to an Accela HPLC system (Thermo Electron) and interfaced to a Thermo Finnigan LTQOrbitrapXL mass spectrometer equipped with an Ion Max electrospray source. Separation of peptides was achieved by a gradient of 5–35% solvent B at 50 μl min^−1^ for 30 min. Solvent B contained 5% water in ACN with 0.1% formic acid. Precursor mass between m/z 350 and 3000 was scanned using the Orbitrap with resolution of 60 000 at m/z 400. LC-MS/MS data files were searched using Sequest search engine through Bioworks (Thermo Electron) and Mascot search engine through in-house Mascot Server (Matrix Science) against bacterial databases. Results from the two search engines were combined using Scaffold Distiller (Proteome Software) for identification of proteins.

### Cross-linking

Chemical cross-linking analysis of Lp6.6-mediated protein complex formation was performed as described (Steiner *et al.*, 2008) with minor modifications. Briefly, isolated OM preparations were incubated with dithiobis(succinimidylpropionate) (DSP) for 2 h on ice according to the instructions of the supplier (Pierce). Cross-linked proteins were incubated in the absence or presence of 5% β-mercaptoethanol for 10 min at 95°C and analysed by immunoblotting with the Lp6.6 antibodies.

### Isolation and infection studies of *lp6.6* mutant and complemented isolates of *B. burgdorferi*

Generation of *B. burgdorferi* mutants were performed using published procedures (Coleman *et al.*, 2008). All primers used for genetic manipulation process are listed in Table S1. The *lp6.6* mutant was constructed by exchanging the *lp6.6* open reading frame with a kanamycin-resistance cassette via homologous recombination. The 5′ and 3′ arms flanking *lp6.6* were amplified using primers P1–P4, then cloned into multiple-cloning sites flanking the kanAn cassette in plasmid pXLF10601 and electroporated into *B. burgdorferi*. Five clones that grew in antibiotic-containing media were further assessed using primers P5–P10 to confirm the desired integration of the *flaBp-kan* cassette into the *B. burgdorferi* genome. One of the *lp6.6* mutants that retained the same set of plasmids as the wild-type isolate was selected for further experiments. Although the construct pXLF10601 (Li *et al.*, 2006) contained an ampicillin resistance marker, no ampicillin-resistant transformants that might result from single-cross-over events were selected. PCR analysis further confirmed the absence of an ampicillin resistance marker in *lp6.6* mutants and no differences in ampicillin sensitivity between the wild-type and mutant spirochetes were observed. For *in vitro* growth analysis, an equal number of wild type and *lp6.6* mutants were diluted to a density of 10^5^ cells ml^−1^ and grown at 23°C or 33°C in BSK-H medium until they reached the stationary phase (10^8^ cells ml^−1^). Aliquots of spirochetes were enumerated using a Petroff–Hausser cell counter under a dark-field microscope.

Complementation of the *lp6.6* mutant was achieved by re-insertion of a wild-type copy of the *lp6.6* gene in the *B. burgdorferi* chromosome as detailed (Li *et al.*, 2007; Yang *et al.*, 2009). Briefly, two DNA inserts encompassing the *lp6.6* open reading frame and the *flaB* promoter were PCR-amplified, fused and cloned into the BamHI and SalI sites of pKFSS1 (Frank *et al.*, 2003) housing a streptomycin-resistance cassette (*aadA*). The *flaB* promoter*–lp6.6* gene fusion construct along with *aadA* cassette was excised from the recombinant pKFSS1 plasmid and inserted into the corresponding restriction sites of the plasmid pXLF14301, which contains the required 5′ and 3′ arms for homologous recombination in the *B. burgdorferi* chromosomal locus *bb0444–0446*. The final construct was sequenced to confirm identity, and 25 μg of the recombinant plasmid was electroporated into the *lp6.6* mutant. One of the five *lp6.6-*complemented clones that was able to grow in antibiotic medium was further selected based on the intended recombination event and the expression of *lp6.6* mRNA and Lp6.6 protein. Analysis of wild-type, *lp6.6* mutant and *lp6.6-*complemented isolates indicated the presence of the same set of endogenous plasmids.

For phenotypic analysis of wild-type and genetically manipulated spirochetes, burdens of viable pathogen in mice and ticks were assessed using qRT-PCR analysis of *flaB* mRNA and normalized against murine or tick *β-actin* genes as described (Coleman *et al.*, 2008; Pal *et al.*, 2008a). To assess whether *flaB* RNA- or *flaB* DNA-based quantitative analyses of spirochete burden produce similar results, qRT-PCR and qPCR analyses were performed using the same infected tissues. C3H mice (three animals per group) were infected with *B. burgdorferi* (10^5^ spirochetes per mouse) and sacrificed at 3 weeks following inoculation, and skin, bladder, heart and joint samples were isolated. In parallel, nymphal ticks (20 ticks per mouse) were allowed to feed on mice infected for 12 days and collected as repleted ticks. Tissues were homogenized in liquid nitrogen, divided into two parts and separately processed for *flaB* DNA-based qPCR (Pal *et al.*, 2004) and *flaB* RNA-based qRT-PCR (Coleman *et al.*, 2008), which produced similar patterns in the differences of the tissue burdens of wild type and *lp6.6* mutants (Fig. S1). Additionally, similar patterns in qPCR- and qRT-PCR-based detection of borrelial burdens were observed when spirochete levels were compared in multiples murine tissues at a later time point, 12 weeks after challenge (data not shown). As mRNA assessment has been shown to be a better surrogate for the detection of viable microbes (Sheridan *et al.*, 1998; Bleve *et al.*, 2003) and previous studies also indicated a general positive correlation between *flaB* mRNA levels and *flaB* DNA-based assessment of pathogen burdens (Hodzic *et al.*, 2003), in subsequent studies, we measured *flaB* RNA using qRT-PCR analysis, rather than analysis of the DNA target. Furthermore, we have determined that deletion of *lp6.6* did not affect *flaB* transcript levels, which are similar in wild-type and mutant *B. burgdorferi* (data not shown). To assess pathogen persistence in the murine host, C3H mice (five animals per group) were infected with *B. burgdorferi* (10^5^ spirochetes per mouse) and sacrificed at 1, 2, 3 and 12 weeks following inoculation. The skin, bladder, heart and joint samples were isolated and stored in liquid nitrogen, and aliquots of blood and spleen tissues were cultured in BSK medium for further assessment of spirochete viability*.* Pathogen burdens were assessed in larval and nymphal ticks (five ticks per mouse) that fed on *B. burgdorferi-*infected mice after 12 days of infection. Naturally infected nymphs were also generated, allowed to feed on naïve mice (five ticks per mouse, five mice per group) and spirochete burdens in ticks were determined by qRT-PCR at 1, 2 and 4 days of feeding. *B. burgdorferi* burdens were assessed in intact salivary glands and the gut at day 2 of feeding, which is a biologically relevant time point of transmission (Piesman *et al.*, 1987; Piesman, 1993) prior to the major blood meal when intact organs can be isolated with less possibility of cross-contamination (Sonenshine, 1993). For other time points, *B. burgdorferi* burdens were assessed in whole ticks without dissection. At 7 days of tick feeding, all the mice were sacrificed, and the skin and heart tissues were isolated and assessed for the spirochete burden by qRT-PCR and culture analysis. To assess if continual expression of *lp6.6* by complemented isolates had any effect on borrelial persistence, parallel groups of C3H mice (six animals) were infected with wild-type or genetically manipulated isolates (10^5^ spirochetes per mouse). After 2 weeks post infection, samples of skin, bladder, heart and joint were isolated and analysed for *lp6.6* transcripts and pathogen burdens as described above.

### Evaluation of disease

*Borrelia burgdorferi*-infected mice were examined for disease by the assessment of joint swelling and histological evaluation of inflammation as detailed earlier (Coleman *et al.*, 2008; Pal *et al.*, 2008a). Ankle joints of each mouse were measured using a precision metric caliper, and development of swelling was monitored weekly. For histology, at least five ankle joints from each group of mice (five animals per group) were collected and fixed in 10% formalin, decalcified and processed for Haematoxylin and Eosin staining. Sections were blindly examined for assessment of histological parameters of *B. burgdorferi-*induced inflammation as detailed (Pal *et al.*, 2008a).

### Statistical analysis

Results are expressed as the mean ± standard error (SEM). The significance of the difference between the mean values of the groups was evaluated by two-tailed Student's *t*-test.
