# Supplementary material for: Borrelia burgdorferi small lipoprotein Lp6.6 is a member of multiple protein complexes in the outer membrane and facilitates pathogen transmission from ticks to mice
Source: Mol Microbiol. 2009 Sep 2;74(1):112–25. doi: 10.1111/j.1365-2958.2009.06853.x (PMC2754595; doi:10.1111/j.1365-2958.2009.06853.x)
Supplement: Supplementary file 1 [file mmi0074-0112-SD1.pdf]

Figure S1

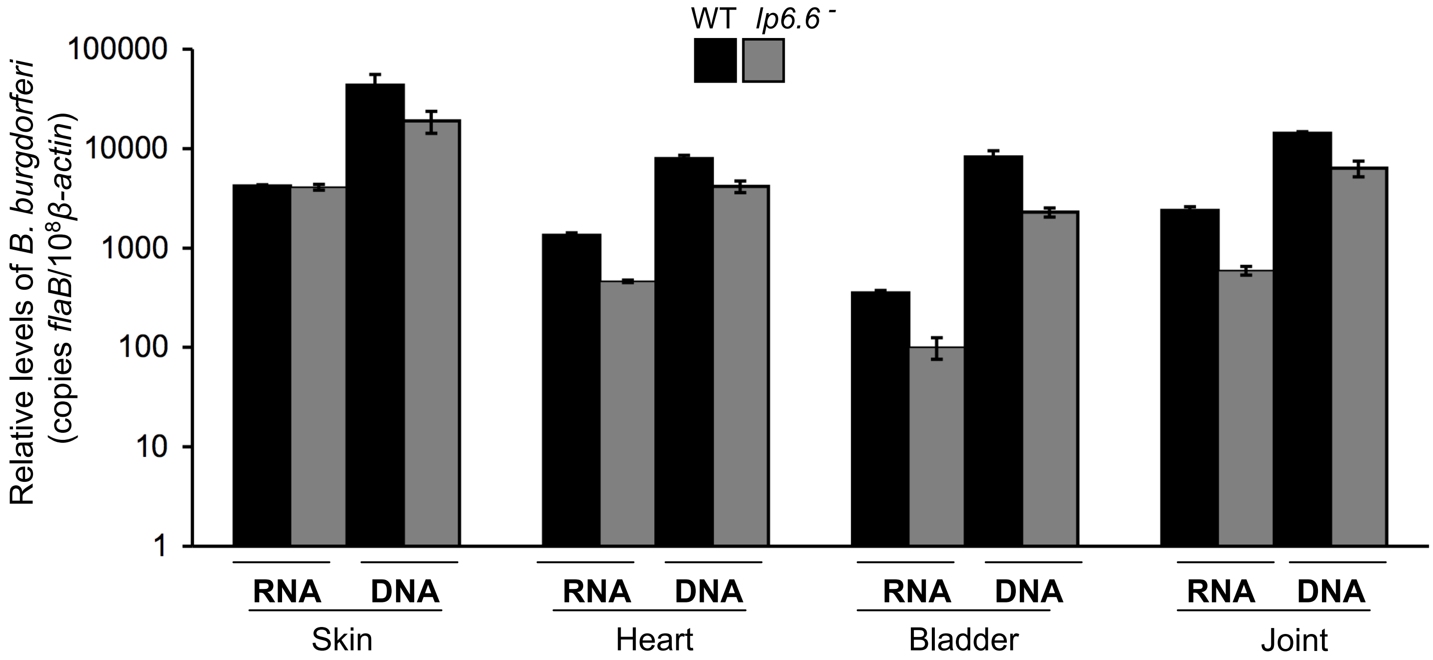

WT
lp6.6 -
100000
10000
1000
100
10
1
Relative levels of B. burgdorferi (copies flaB/10^8 β-actin)
RNA
DNA
Skin
RNA
DNA
Heart
RNA
DNA
Bladder
RNA
DNA
Joint

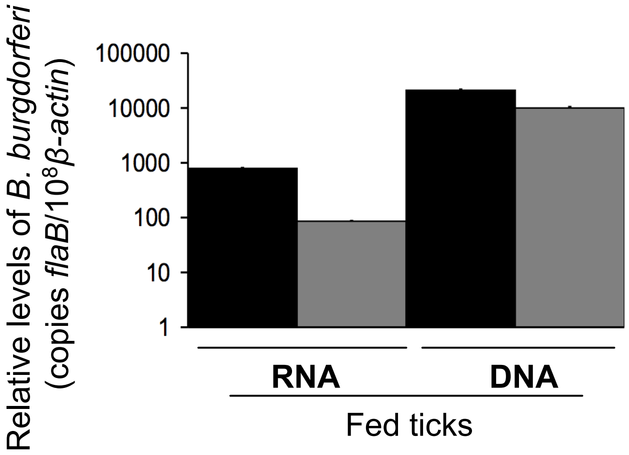

Relative levels of B. burgdorferi (copies flaB/10^8 β-actin)
100000
10000
1000
100
10
1
RNA
DNA
Fed ticks

Supplementary Table S1. Oligonucleotide primers used in the current study.

Sequence (5'→ 3')	Purpose
TTGCTGATCAAGCTCAATATAACCA	Forward primer for Quantitative PCR of *B. burgdorferi flaB*
TTGAGACCCTGAAAGTGATGC	Reverse primer for Quantitative PCR of *B. burgdorferi flaB*
AGAGGGAAATCGTGCGTGAC	Forward primer for Quantitative PCR of mouse *β-actin*
CAATAGTGATGACCTGGCCGT	Reverse primer for Quantitative PCR of mouse *β-actin*
GGTATCGTGCTCGACTC	Forward primer for Quantitative PCR of tick *β-actin*
ATCAGGTAGTCGGTCAGG	Reverse primer for Quantitative PCR of tick *β-actin*
TCC*CCGCGG*AGCTATTCTTAAAGTATAGC	Primer P1, 5' PCR of the left arm for constructing *lp6.6* mutant. A *SacII* site (italicized) is attached for cloning.
TCC*CCCGGG*TTATTCTCCTTCTTTAAAAC	Primer P2, 3' PCR of the left arm for constructing *lp6.6* mutant. A *SmaI* site (italicized) is attached for cloning.
CCC*AAGCTT*TGTAAATTAATTTAAATAC	Primer P3, 5' PCR of the left arm for constructing *lp6.6* mutant. A *HindIII* site (italicized) is attached for cloning.
GG*GGTACC*ACTGTGCTAATTTTGATTTC	Primer P4, 3' PCR of the left arm for constructing *lp6.6* mutant. A *KpnI* site (italicized) is attached for cloning.
AAGAAAGCATATGACAAAATTAATGTACGCTAT	Primer P5, 5' PCR primer for the detection of *lp6.6* locus
ACGCGTCGACTTACTTTTTCATTGACTTTGTC	Primer P6, 3' PCR primer for the detection of *lp6.6* locus
GGTTGCATTCGATTCCTGTT	Primer P7, 5' PCR primer for the detection of intended integration of *pFlab-Kan* cassette in *lp6.6* locus
ATTCCGACTCGTCCAACATC	Primer P8, 3' PCR primer for the detection of intended integration of *pFlab-Kan* cassette in *lp6.6* genomic locus
CGTGAGGCAGTAGGTCCAAT	Primer P9, downstream PCR primer to confirm deletion of *lp6.6* locus
GGGGTACCTTTTTCTCTTGCGATCTAAAC	Primer P10, upstream PCR primer to confirm deletion of *lp6.6* locus
GGCTCACAAAACCAGTATGGA	Forward primer for RT-PCR of *bba61*
GCAAATTGCCTTCTTTGTCG	Reverse primer for RT-PCR of *bba61*
TGTTGCTTGCGAAACTACAAG	Forward primer for Quantitative PCR or RT-PCR of *lp6.6*
TGTCTGGCATTGACTTCATCA	Reverse primer for Quantitative PCR or RT-PCR of *lp6.6*
GCAGCCTTGACGAGAAAAAC	Forward primer for Quantitative PCR of *ospA*
TGTCAGCTTTTACGCCTTCA	Reverse primer for Quantitative PCR of *ospA*
CG*GGATCC*TGCGAAACTACAAGAATTTCAG	Forward primer for recombinant Lp6.6 production. A *BamHI* site (italicized) is attached for cloning.
CG*GAATTC*TTTTTCTAAATCAAAATAATAAATAGTT ATTAG	Reverse primer for recombinant Lp6.6 production. An *EcoRI* site (italicized) is attached for cloning.
CG*GGATCC*GTCTGTCGCCTCTTGTGGCTTC	Forward primer for *flaB* promoter. A *BamHI* site (italicized) is attached for cloning.
GTATTATGATTGATAAT*CATATG*TCATTCCTCCATG	Reverse primer for *flaB* promoter. A *NdeI* site (italicized) is attached for cloning.
AAGAAAG*CATATG*ACAAAATTAATGTACGCTAT	Forward primer for constructing the *lp6.6* complemented isolate. A *NdeI* site (italicized) is attached for the purpose of cloning.
ACGC*GTCGAC*TTACTTTTTCATTGACTTTGTC	Reverse primer for constructing the *lp6.6* complemented isolate. A *SalI* site (italicized) is attached for the purpose of cloning.
